# Acute Arthritis Treated with Antistreptococcic Serum

**Published:** 1905-10-07

**Authors:** 


### ACUTE ARTHRITIS TREATED WITH ANTISTREPTOCOCCIC SERUM.

The patient was a woman of 51 years, and was under the care of Dr. H. D. Rolleston[1] at St. George's Hospital. The disease was of acute onset, one month before admission to the hospital, and the knees were the joints first afflicted. Afterwards pain and swelling appeared in the wrists, ankles, and fingers. The hands showed ulnar deflection at the wrists, and the inter-philangeal and metacarpo-phalangeal joints were enlarged and red. The temperature on admission was 102.6. In short, the condition was identical with the description given of "acute rheumatoid arthritis." There was no history of gonorrhœa, and no other possible source of infection could be discovered, either in the mouth or elsewhere. Treatment at first included a liberal diet and the administration of iodide of iron. This was continued for five days, but failed to produce any improvement in the temperature, the pulse (which ranged from 90 to 120), or in the state of the joints. Salicylate of sodium was therefore ordered, and was continued for several days, being first given in an alkaline mixture, and subsequently with iron. Here again no substantial benefit could be noted, and the pyrexia continued unabated. On November 8th, 10c.c. of polyvalent antistreptococcic serum prepared at the Pasteur Institute were injected, with the result that within 36 hours the temperature fell to 99°, and remained between that level and 99.8° until the 14th, when 10cc. of the polyvalent serum prepared at the Lister Institute were injected. This was followed by a marked reaction, the temperature on the 16th rising to 103°, but afterwards falling gradually to normal. At the same time the condition of the joints improved greatly. On December 2nd a third injection of serum was given, and was again followed by reaction, the temperature reaching 102.6°. In the course of a few days it fell to normal, and at the expiry of a month the patient was able to leave the hospital for Bath, with great improvement in the condition of the joints and with considerable ability to use them. An urticarial rash and some albuminuria were noted subsequent to the use of the serum. Both symptoms had completely disappeared before the patient left the hospital.

[1] Lancet, September 30, 1905.

